# Response Strategies against Meningitis Epidemics after Elimination of Serogroup A Meningococci, Niger

**DOI:** 10.3201/eid2108.141361

**Published:** 2015-08

**Authors:** Halima Boubacar Maïnassara, Juliette Paireau, Issa Idi, Jean-Paul Moulia Pelat, Odile Ouwe Missi Oukem-Boyer, Arnaud Fontanet, Judith E. Mueller

**Affiliations:** Université Pierre et Marie Curie, Paris (H.B. Maïnassara, J. Paireau);; Institut Pasteur, Paris, France (H.B. Maïnassara, J. Paireau, A. Fontanet, J.E. Mueller);; Centre de Recherche Médicale et Sanitaire, Niamey, Niger (H.B. Maïnassara, I. Idi, J.-P.M. Pelat, O.O.M. Oukem-Boyer);; Chaire Santé et Développement, Paris (A. Fontanet);; Ecole des Hautes Études en Santé Publique, Rennes, France (J.E. Mueller)

**Keywords:** Meningitis, epidemics, Niger, surveillance, bacteria, vaccines

## Abstract

To inform epidemic response strategies for the African meningitis belt after a meningococcal serogroup A conjugate vaccine was introduced in 2010, we compared the effectiveness and efficiency of meningitis surveillance and vaccine response strategies at district and health area levels using various thresholds of weekly incidence rates. We analyzed reports of suspected cases from 3 regions in Niger during 2002–2012 (154,392 health area weeks), simulating elimination of serogroup A meningitis by excluding health area years with identification of such cases. Effectiveness was highest for health area surveillance and district vaccination (58–366 cases; thresholds 7–20 cases/100,000 doses), whereas efficiency was optimized with health area vaccination (5.6–7.7 cases/100,000 doses). District-level intervention prevented <6 cases (0.2 cases/100,000 doses). Reducing the delay between epidemic signal and vaccine protection by 2 weeks doubled efficiency. Subdistrict surveillance and response might be most appropriate for meningitis epidemic response after elimination of serogroup A meningitis.

## Abstract 2

Surveillance and epidemic vaccine response would be most effective at the health area level.

For several decades, epidemic meningitis has been a major health problem in the African meningitis belt. *Neisseria meningitidis* serogroup A (NmA) has been responsible for most localized epidemics or epidemic waves, and other meningococcal serogroups occasionally caused epidemics (*1**,**2*). After the introduction of an NmA conjugate vaccine (PsA-TT, MenAfrivac; Serum Institute of India Ltd., Hadapsar, Pune, India), implemented since 2010 in mass campaigns focused on persons 1–29 years of age, no epidemics caused by NmA have occurred in countries where the vaccine is administered (i.e., vaccinated countries). Seasonal hyperendemicity continues to occur during the dry season because of meningococci and pneumococci in similar proportions (*3*), and NmA has been identified only exceptionally (*4**,**5*). So far, epidemic control measures have consisted of reactive vaccination campaigns in epidemic districts by using serogroup A/C or A/C/W polysaccharide vaccines combined with adapted treatment protocols. To detect epidemics, national routine surveillance of suspected cases of acute bacterial meningitis has been conducted in all meningitis belt countries according to World Health Organization (WHO) guidelines (*1*), although the recommendation of splitting large districts (>100,000 inhabitants) into subdistricts is not always followed. Districts notifying weekly incidences of 5 cases/100,000 persons were considered in alert, and those notifying weekly incidences of 15 cases/100,000 persons were considered in epidemic (*6*); however, specific conditions enabled declaration of an epidemic at a threshold of 10 cases/100,000 persons.

Since PsA-TT was introduced in 2010, NmA incidence has been substantially lower than historical levels, and no replacement by other serogroups has been observed (*7*). Consequently, the overall incidence of suspected meningitis cases has declined in all vaccinated countries, and established surveillance and vaccination strategies might no longer be appropriate. Epidemic detection and response remains important because serogroups W and X have epidemic potential (*1**,**2**,**4**,**8**,**9*). A polysaccharide vaccine is available against *N. meningitidis* serogroup W (NmW); vaccines for serogroup X (NmX) are under development. NmA epidemics might continue to occur, requiring mass vaccination campaigns with PsA-TT.

One approach to adapting epidemic response strategies to the changing epidemiology is to lower the weekly incidence thresholds to <10 cases/100,000 persons, which would increase the risk for an increased number of false alerts in small districts. Another approach is to analyze surveillance data at a finer spatial scale than district level. As in Burkina Faso (*2*,*10*) and Niger (*11*), epidemics of any serogroup usually are highly localized in few neighboring health centers, whereas most health centers in the district in question remain (hyper-)endemic. District-level incidences are therefore diluted and may hide epidemic activity. Consequently, surveillance at the health center level could detect epidemics earlier and enable targeting of reactive vaccination, making the overall strategy more effective and efficient. Early epidemic detection through surveillance at the health center level could increase the effectiveness and efficiency of response strategies, particularly in the anticipated situation of eliminated NmA meningitis and overall reduced meningitis incidence.

Therefore, during 2002–2012, we evaluated the effectiveness and efficiency of surveillance and vaccine response strategies in Niger using various epidemic thresholds and comparing health area and district intervention. Our analysis was based on surveillance data of suspected and confirmed cases and considered 2 scenarios: the historical situation before PsA-TT introduction (PsA-TT was introduced in Niger in 3 phases: the first in August 2010, the second in November 2010, and the third in October 2011) and a simulation of NmA elimination.

## Methods

### Databases

**Figure 1:**
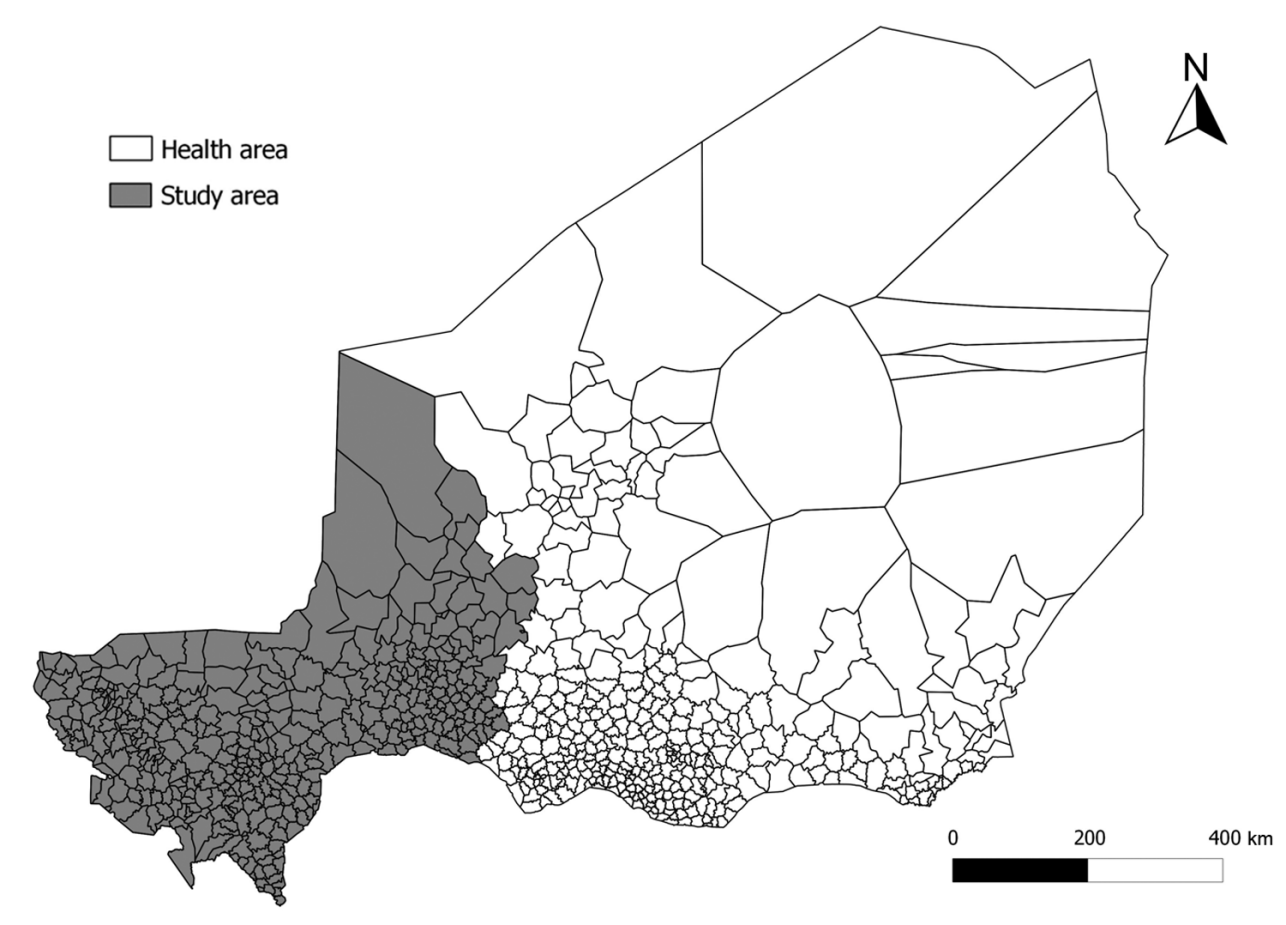
Location of the study area, Niger. This area comprises the 379 health areas in 3 regions (Tahoua, Tillabery, and Dosso).

The surveillance data used for this analysis were collected during 2002–2012 in Niger. For national routine surveillance, all health centers in Niger report suspected meningitis case counts each week to health district administration, where data are aggregated to a district case count and forwarded to the national level for reporting (Technical Appendix Figure 1) (*12*,*13*). To analyze response strategies at the health area level, we retrieved the original health center counts of suspected meningitis cases from health district administrations and compiled them in a new database. A health area is a geographic area that encompasses all villages served by the same health center, which is an exclusive association. We assessed the completeness of this health area database of suspected cases by comparing the resulting weekly district case counts with those in the national surveillance reports. On the basis of the completeness of this health area–level database (online Technical Appendix), we selected 3 regions—Tahoua, Tillabery, and Dosso—for further analysis (Figure 1). Together, these regions had 7,648,150 inhabitants during 2012 (48% of Niger’s population), 19 health districts (47%), and 373 health areas (51%). Niger used *Haemophilus influenzae* type b vaccine since 2006 but uses no pneumococcal vaccine in the widened vaccine program.

Data on confirmed cases came from countrywide surveillance based on PCR (*11*). For this surveillance, we requested that all cerebrospinal fluid samples from persons with suspected meningitis in the health centers be sent to the Centre de Recherche Médicale et Sanitaire (Niamey, Niger) for testing by multiplex PCR (*14*). After merging with the study database of suspected cases, we obtained the combined information about suspected and confirmed meningococcal case counts for every health area and week.

**Figure 2:**
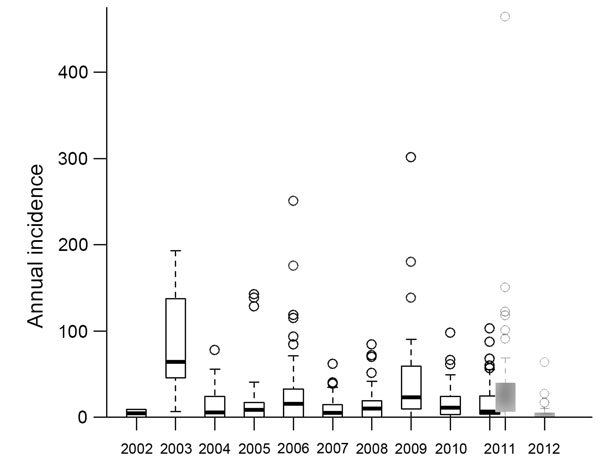
Annual incidences of suspected meningitis per 100,000 inhabitants in health areas before and after introduction of PsA-TT (in 2010) in a database simulating elimination of serogroup A meningococci. Tahoua, Tillabery, and Dosso regions, Niger, 2002–2012. The period before PsA-TT (2002–October 2011, last phase of vaccination during November 2011) (white bars) comprises 433 health area years. The period after PsA-TT (October 2010–December 2012, first phase of vaccination during September 2010) (gray bars) comprises 98 health area years. Excluded were health area years during which at least 1 serogroup A case was detected or for which no serogroup information was available. The number of health areas in this database varied by year from 2 (2002) and 10 (2003) to 126 (2011). Each circle corresponds to annual incidence in a health area. Dark lines are parts of the boxplot, as follows: for 2003 (complete boxplot), the first line corresponds to the minimal annual incidence of health area, the second line corresponds to the limit of the first quartile and the third (darkest) corresponds to the median. The space between the second third lines corresponds to and second quartile. The fourth line is the limit of the third quartile and the last line is the limit of the fourth quartile of annual health area incidence. PsA-TT, serogroup A meningococcal conjugate vaccine (MenAfrivac [Serum Institute of India Ltd., Hadapsar, Pune, India]).

Using these data, we prepared a database simulating the situation after NmA elimination by excluding all health area years with at least 1 NmA case or without any laboratory information. A health area year corresponded to a health area that appears in the annual reporting file, so that in our analysis health area years represented all the health areas that appear in annual reporting files during the study period. Only 1 NmA case was identified in the study region after PsA-TT introduction. To validate the representativeness of this database for meningitis epidemiology after NmA elimination, we compared the distribution of annual incidences between health areas before and after PsA-TT introduction (Figure 2). To prepare the database simulating the situation after NmA elimination, we took into account the exact week of district vaccination, which was conducted in 3 phases: September 2010, December 2010, and November 2011. The Niger national ethics committee approved this research (no. 014/2012/CCNE).

### Statistical Analysis

The Institut National de la Statistique provided the number of inhabitants in each village according to the 2001 national census. We aggregated the villages’ populations at the health area level and applied a mean annual growth rate of 3.3% (*15*). We calculated weekly incidence rates (WIR) as the weekly number of cases per 100,000 inhabitants and annual incidence (AI) as the number of cases per 100,000 inhabitants during an epidemiologic year. To compile cases belonging to the same meningitis season (approximately December–May), we defined an epidemiologic year as July 1 of calendar year *n-1* through June 30 of calendar year *n*.

We evaluated the effectiveness and efficiency of surveillance and vaccine response strategies by calculating the number of potentially vaccine-preventable cases and the number of vaccine doses needed per epidemic. These calculations were repeated with the database including NmA to determine whether the situation differed from that simulating NmA elimination.

Using receiver–operator curves as previously described (*10*), we chose candidate epidemic thresholds to optimize the detection of annual incidences that are above the 95th or 97.5th percentiles of health area annual incidences in the databases (online Technical Appendix Figure 3). An epidemic was defined as weekly incidence rate in a health area that exceeded the corresponding threshold for at least 1 week.

**Figure 3:**
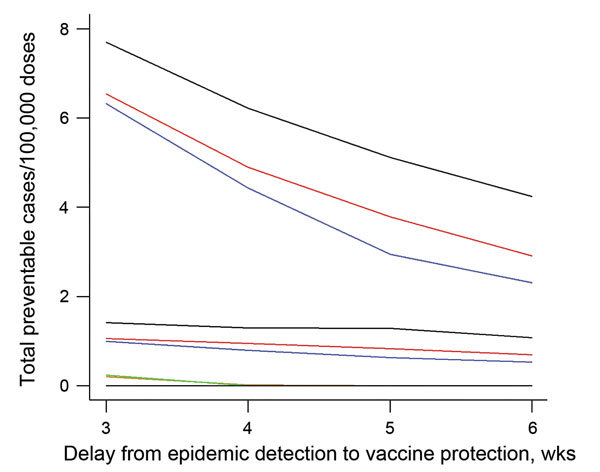
Comparison of preventable meningitis cases per 100,000 vaccine doses, given different surveillance and meningococcal vaccine response strategies, in a situation simulating elimination of *Neisseria meningitides* serogroup A, Tahoua, Tillabery, and Dosso regions, Niger, 2002–2012. Three, 4, 5, and 6 weeks delay were considered between epidemic detection and effective vaccine protection. The strategies were surveillance and vaccination at health area level (health area–health area, top 3 lines), surveillance at health area level combined with vaccination of the district (health area–district, middle 3 lines), and surveillance and vaccination at district level (district–district, bottom 3 lines). For the health area–health area and health area–district strategies, the black line indicates preventable cases/100,000 vaccine doses at an incidence threshold of 7 cases/100,000 inhabitants; red line, threshold of 10 cases/100,000 inhabitants; and blue line, threshold of 15 cases/100,000 inhabitants. For the district–district strategy, the green line indicates preventable cases/100,000 vaccine doses at an incidence threshold of 4 cases/100,000 inhabitants; orange line, threshold of 2 cases/100,000 inhabitants; and black line, threshold of 7 cases/100,000 inhabitants.

We evaluated 3 strategies: 1) surveillance (including data analysis) and vaccination at health area level; 2) surveillance at health area level and vaccination of the entire district; and 3) surveillance and vaccination at district level. We approximated the number of potentially vaccine-preventable cases (Nvp) as

Nvp = N*3w* × P*Nm* × VC × VE,

where N*3w* is the number of suspected cases in the surveyed health area or district from 3 weeks after the threshold is exceeded (assuming that campaign implementation and effective protection from vaccine antibody would take at least 3 weeks after signal detection); P*Nm* is the percentage of suspected cases confirmed as Nm, estimated as 50% in both epidemic and endemic periods, on the basis of previous surveillance reports (*16*); VC is the expected vaccine coverage during a mass campaign in response to an outbreak, estimated at 80% (*5*); and VE is the expected vaccine effectiveness, estimated at 80%. Because no data for NmW and future NmX vaccines (polysaccharide or conjugate) were available to inform this assumption, we used the available vaccine effectiveness data of NmA polysaccharide vaccine (*17*) and NmW and NmA polysaccharide vaccine combined (*18*). This assumption might be conservative, in particular because conjugate vaccines will be used for epidemic response against non-A serogroups (*19*). We calculated the number of vaccine doses needed per epidemic as the age group 1–29 years of the total population, which was 74% for the study regions according to census data (*20*); and the total number of preventable cases in the population of interest for 100,000 vaccine doses. To evaluate the sensitivity of our estimates to a longer delay from signal detection to effective vaccine protection, we varied this delay from 3 weeks to 6 weeks. We used the rate ratio test to compare annual incidences assuming that they follow a Poisson distribution.

All analyses were performed in R software version 2.15.2 (http://www.R-project.org). Maps were created with QGIS software version 1.8.0 (http://qgis.osgeo.org).

## Results

### Descriptive Epidemiology

Our study comprised 154,392 weekly health area reports. Among the reports were 14,921 suspected cases, of which 13,620 (91.3%) occurred during calendar weeks 1–20, corresponding to the meningitis season (Technical Appendix Figure 2). At district level, median AI was 10.5 cases per 100,000 inhabitants; WIR peaked at 10 cases per 100,000 during most years, and maximum incidences were 50 in Tillabery region during 2006 and 30 in Tahoua region during 2008. At health area level, median AI was 0 cases per 100,000 inhabitants, with a maximum of 1,384 for health areas in Tillabery, 959 in Tahoua, and 777 in Dosso. WIR peaked at 613 cases per 100,000 inhabitants.

In the database simulating NmA elimination, median district-level AI was 10.5 cases (range 0–166) per 100,000 inhabitants; WIR peak was a median of 2 (maximum 50) per 100,000. Median health area–level AI was 10 cases (maximum 464) per 100,000 inhabitants; WIR peaked at 172 per 100,000.

In the database simulating NmA elimination, median district-level AI was 13 cases (range 0–166) per 100,000 inhabitants before and 4 (range 0–41) per 100,000 after PsA-TT introduction. Median health area–level AI was 10 cases (range 0–302) per 100,000 inhabitants before and 9 (range 0–464) per 100,000 after PsA-TT introduction (Figure 2). Mean health area–level AI was 21 cases per 100,000 inhabitants before and 26 per 100,000 after PsA-TT introduction (p = 0.43). The similarity of these numbers suggests that the simulation of NmA elimination based on data before PsA-TT introduction is representative of the situation after PsA-TT introduction.

### Evaluation of Strategies

In the database simulating NmA elimination, the thresholds providing best sensitivity and specificity to detect AI beyond the 95th and 97.5th percentiles were, at health area level, WIR of 20 and 15 cases per 100,000 inhabitants, respectively, and at district level, WIR of 4 per 100,000, for both percentiles (Technical Appendix Figures 3, 4).

The total number of epidemics requiring a response during the 11-year period in the study area varied from 3 to 15 for district surveillance and from 49 to 233 for health area surveillance. The number of total vaccine-preventable cases varied from 0 to 6 (thresholds 2–7/100,000) with district surveillance and vaccination, from 27 to 213 cases (thresholds 7–20/100,000) with health area surveillance and vaccination, and from 58 to 366 cases with health area surveillance combined with district-level vaccination (thresholds 7–20/100,000) (Table 1). Although the latter strategy was most effective, it required the largest number of vaccine doses (8.7–25.7 million, depending on the threshold), whereas the 2 other strategies consumed a similar number of doses (≈0.5–3 million). Efficiency was lowest for district surveillance (<0.24 vaccine-preventable cases/100,000 doses) and highest for health area surveillance and vaccination (7.7 cases/100,000 doses). Overall, the difference in effectiveness or efficiency for different geographic levels of intervention was greater than for any change of threshold. Among individual health areas, the effect of any vaccine response followed a skewed distribution: the median number of preventable cases per 100,000 doses was close to 0 in most scenarios, and a maximum of 178 cases were preventable.

**Table 1:** Comparison of estimated vaccine-preventable meningitis cases using different strategies of surveillance and meningococcal vaccine response in a situation simulating elimination of meningococcal serogroup A, Tahoua, Tillabery and Dosso regions, Niger, 2002–2012*

Strategy, surveillance–vaccination	Threshold	Total no. epidemic signals	Population affected by signal	Vaccine doses in persons 1–29 y†		Vaccine-preventable cases‡					
						No.			Per 100,000 cases		
				Total	Median	Total	Median		Total	Median	Range
Health area–health area	7	233	3,741,116	2,768,426	9,721	213	0.32		7.70	2.31	0–178.35
	10	165	2,453,831	1,815,835	9,346	119	0.32		6.54	2.38	0–178.35
	15	80	1,142,888	845,737	8,817	53	0.00		6.32	0.00	0–178.35
	20	49	641,378	474,620	8,272	27	0.00		5.60	0.00	0–41.02
Health area–district	7	233	35,297,443	25,866,453	258,625	366	0.96		1.42	0.44	0–14.38
	10	165	31,304,108	23,165,040	259,647	246	2.80		1.06	0.27	0–13.23
	15	80	17,062,861	12,626,517	284,407	126	0.96		1.00	0.30	0–7.87
	20	49	11,757,953	8,700,885	287,661	58	0.80		0.66	0.23	0–3.36
District–district	2	15	4,053,961	2,999,931	216,403	6	0.00		0.20	0.00	0–1.56
	4	8	2,710,118	2,005,487	269,749	5	0.00		0.24	0.00	0–1.56
	7	3	936,406	692,940	250,957	0	0.00		0.00	0.00	0

*Weekly incidence rate thresholds (cases/100,000 inhabitants were selected on the basis of best performance (sensitivity and specificity) to identify years of high annual incidence (531 health area years). The 3 regions had 7.5 million inhabitants. †Case-patients <2 y of age were not excluded. ‡Median, across all health areas or districts with epidemic signal; total: for entire study area of 3 regions (population 7,648,128); mean: mean per signal (health area or district level).

When the assumed delay between epidemic signal and effective protection from vaccine antibody increased from 3 to 5 weeks, efficiency was halved, and the difference among the strategies was halved. At 6 weeks’ delay, health area surveillance and vaccination strategy remained the most efficient (2–5 cases/100,000 doses). District surveillance and vaccination at any threshold failed to prevent cases in a delay of >4 weeks (Figure 3).

When we evaluated data within the entire database for 2002–2012, including health areas with NmA identification in a year (Table 2), the total number of epidemics requiring response varied from 18 to 92 for district activity and from 232 to 844 for health area activity. The number of total vaccine-preventable cases varied less with geographic level than with threshold (overall range 652–3,739 cases). By contrast, efficiency varied more strongly with geographic level than with threshold; health area surveillance and vaccination prevented 21–26 cases per 100,000 doses, compared with 14–16 cases for district intervention.

**Table 2:** Comparison of estimated vaccine-preventable meningitis cases using different strategies of surveillance and meningococcal vaccine response before introduction of PsA-TT, Tahoua, Tillabery, and Dosso regions, Niger, 2002–September 2011

Strategy, surveillance–vaccination	Threshold*	Total no. epidemic signals	Population affected by signal	Vaccine doses in persons 1–29 y		Vaccine preventable cases					
						No.			Per 100,000 doses		
				Total	Median	Total	Median		Total	Median	Range
Health area–health area	7	844	14,742,136	10,909,180	10,051	2,282	0.64		20.92	5.18	0–595.34
	10	679	11,567,626	8,560,043	10,081	1,924	0.64		22.48	5.41	0–595.34
	15	469	7,849,057	5,808,302	9,587	1,332	0.64		22.93	5.59	0–482.10
	20	358	5,791,671	5,791,671	9,307	1,087	0.64		25.36	6.14	0–482.10
	30	235	3,719,950	3,719,950	8,919	717	0.64		26.05	8.25	0–482.10
Health area–district	7	844	47,090,036	34,846,626	250,877	3,739	4.64		10.73	3.42	0–70.20
	10	679	44,361,151	32,827,251	251,352	3,353	5.76		10.21	2.55	0–70.20
	15	469	36,118,246	26,727,502	251,574	2,689	4.80		10.06	2.32	0–69.16
	20	358	29,902,138	22,127,582	250,956	2,276	4.48		10.29	1.75	0–58.53
	30	235	23,115,711	17,105,626	244,380	1,612	2.88		9.43	1.27	0–58.53
District–district	2	92	29,251,073	21,645,794	244,595	3,103	12.00		14.33	6.05	0–61.19
	4	68	21,069,851	15,591,689	244,595	2,343	13.60		15.03	5.35	0–61.19
	7	44	14,075,282	10,415,708	252,666	1,483	17.44		14.24	7.93	0–46.79
	10	18	6,021,142	4,455,645	252,049	652	18.40		14.64	9.74	0–35.28
	15	18	6,021,142	4,455,645	252,049	652	18.40		15.64	9.74	0–35.28

*Weekly incidence rate thresholds were selected on the basis of best performance (sensitivity and specificity) to identify years of high annual incidence (2,534 health area years). The 3 regions had 7.5 million inhabitants.

## Discussion

Using meningitis surveillance data from Niger during an 11-year period, we estimated that after elimination of NmA from the meningitis belt surveillance and epidemic vaccine response would be more effective and efficient if conducted at the health area level rather than at the district level, as is currently done in most meningitis belt countries. In Niger, health areas typically have a median population of 14,440 inhabitants, ≈20-fold smaller than districts (median 295,200 inhabitants). Although the WHO epidemic response guidelines recommend splitting large districts (>100,000 inhabitants) into subdistricts for data analysis (*12*), surveillance data during the last decade have been analyzed at no higher resolution than district level in most instances. Our analysis confirms that before NmA elimination and before PsA-TT introduction, the district-level strategy yielded good effectiveness and efficiency comparable with health area–level analysis of surveillance data, while it minimized the number of individual events requiring a response. In light of these data, we recommend that more countries explore the expected impact and feasibility of subdistrict-level surveillance and vaccination after NmA elimination.

Although meningococcal polysaccharide vaccines have been used in the meningitis belt for several decades, including occasionally for prevention strategies, no evidence has been found of a major effect on meningococcal epidemiology beyond outbreak control. By contrast, PsA-TT, a protein-conjugate vaccine, appears to have eliminated NmA disease in the region, probably because conjugate vaccines induce much higher antibody levels than do polysaccharide vaccines (*21*), leading to mucosal immunity and persisting longer. Mucosal immunity leads to reduced transmission in vaccinees and to indirect protection against infection and disease for the entire population. PsA-TT mass campaigns focusing on persons in a large range of ages have achieved high coverage in Niger (*22*), such that circulation of NmA has been drastically reduced and NmA disease eliminated. This interpretation is supported by evidence from Chad (*23*) and Burkina Faso (*24*). Further surveillance is needed to show the duration of this elimination, but if a long-term vaccination strategy that includes protection of new birth cohorts were established, this situation could be sustainable. Consequently, surveillance and epidemic response for meningitis outbreaks of other etiologies such as other meningococcal serogroups or pneumococcus will need to be adapted for the long term.

In our analysis, in the event of a health area–level epidemic signal, vaccinating the entire district achieved higher effectiveness than did focusing on the epidemic health area, whereas the latter appeared more efficient relative to the number of vaccine doses required. An intermediate strategy might involve ring vaccination of health areas neighboring the epidemic area. In any case, because vaccine campaigns would be launched in small health areas after as few as 2 or 3 cases, the choice of exact strategy will need to account for country-specific factors, such as logistic and economic constraints and preferences. The availability of laboratory confirmation will provide further arguments.

Given the short duration of individual meningococcal meningitis epidemics, rapid decision making and implementation of mass campaigns is essential to maximizing the impact of the epidemic response. In our analysis, decreasing the delay from signal detection to effective vaccine protection by 2 weeks doubled efficiency (and number of cases prevented). The updated version of the WHO guidelines further emphasizes this aspect. However, several relatively fixed factors make it challenging to reduce the currently observed delay, such as weekly rhythm of data analysis, storage of vaccines at a central location in a country or even at international level, with corresponding administrative and transport delay, and time required to mobilize vaccination teams. More reactive signal detection in health areas, possibly combined with decentralized vaccine management at the regional level, is therefore an interesting option for improving the impact of reactive vaccination.

Our analysis indicated that several hundred cases were preventable during the 11-year period; however, among individual epidemics, a median of near 0 cases were prevented per 100,000 doses, and a maximum of 180 cases were prevented. This finding indicates that in many epidemic health areas, no cases would be prevented by reactive vaccination, but a large benefit would appear in a few situations, probably where the outbreak is longer. Our analysis overestimates current potential vaccine impact because a proportion of the outbreaks resulted from NmX, for which no vaccine exists. Other assumptions may lead to overestimation or underestimation of vaccine-preventable cases, but regardless, the overall expected impact from reactive vaccination after NmA elimination may be limited. An exception might be a strong increase in the percentage of Nm among suspected cases that result from emergence of non-A serogroups that might increase the effectiveness of any strategy.

Bringing data resolution for meningitis surveillance to the health area level would require relatively few additional means: throughout the meningitis belt, suspected case data are collected in health centers and transmitted in aggregate form by districts to the national level so that a lower degree of aggregation would accelerate data availability. Routine procedures for data management and analysis would need to be slightly modified, but analyses would consist mostly in automated signal detection. Estimates of the referring population usually are available from national census data of villages and health intervention planning. However, several countries may encounter difficulties related to the availability of reliable and up-to-date demographic estimates at health center level. In this case, countries could consider and test other subdistrict resolutions of data analysis. In contrast, substantial efforts would be needed to bring vaccine response to the health area level because bringing response to this level would require decentralization of vaccine management, including prior distribution of vaccine to regions or districts. As a positive side effect, bringing vaccine response to the health area level would further speed up reactive vaccination and thus increase the effectiveness of outbreak response. More focused vaccination would require fewer human resources than do districtwide campaigns and therefore reduce health care disruption, a major shortcoming of mass campaigns. In any instance, countries will need to explore feasibility of subdistrict surveillance and vaccination, identify optimal resolutions, and base decisions on their means and priorities.

Our analysis has several limitations, related mainly to the nature of the available data. We selected only 3 regions in Niger, for which health area counts of suspected case corresponded best with district counts documented nationally. Our analysis therefore did not include densely populated regions at the border with Nigeria, which have regularly reported epidemics, including epidemics caused by NmW or NmX. Furthermore, reporting and sample submission might have been more exhaustive and precise during epidemic situations, leading to overestimation of incidence and of the contribution of epidemic agents. When preparing the database simulating NmA elimination, we excluded health areas without any laboratory confirmation in a year, which includes health areas without any suspected cases. That most health areas report at least 1 case per year probably has led to an overestimation of the median annual incidence after NmA elimination. However, this fact does not affect our estimation of effectiveness and efficiency because these health areas would not have contributed to any epidemic signal. Finally, we calculated incidence rates using the 2001 population census data, to which we applied a constant population growth rate, instead of accounting for potential spatio-temporal variations in migration, ethnic factors, and fecundity rate. We believe that the differences we found among surveillance strategies are sufficiently robust to justify revisiting the current surveillance strategies. By combining routine case reporting and confirmed case incidence at health area level, our database provides a rare opportunity for investigating alternative surveillance strategies around the change in serogroup. Other countries should analyze suspected case incidence after PsA-TT introduction at health center level over the next few years to validate our findings and to inform optimal epidemic surveillance and response strategies.

Our evaluation highlights the value of continuous meningitis surveillance over longer periods and at subdistrict resolution and has the potential to guide future recommendations for reactive vaccination. Also, given a possibly limited absolute impact of reactive vaccination, the results recall that improved prevention strategies should be developed to reduce the effects of acute bacterial meningitis in the African meningitis belt, for example using multivalent meningococcal and pneumococcal vaccines in extended target age groups.

**Technical Appendix.** Data transmission and collection for suspected meningitis case reporting, Niger; evaluation of completeness of the health center database; weekly incidence rates of suspected meningitis cases by health district in 3 regions, Niger, 2002–2012; performance of epidemic threshold definitions in detecting elevated annual meningitis incidences at the health area and district levels, in a situation simulating elimination of meningococcal serogroup A meningitis in 3 regions, Niger, 2002–2012.
