# Supplementary material for: Response Strategies against Meningitis Epidemics after Elimination of Serogroup A Meningococci, Niger
Source: Emerg Infect Dis. 2015 Aug;21(8):1322–9. doi: 10.3201/eid2108.141361 (PMC4517723; doi:10.3201/eid2108.141361)
Supplement: Supplementary file 1 — Technical Appendix. Data transmission and collection for suspected meningitis case reporting, Niger; evaluation of completeness of the health center database; weekly incidence rates of suspected meningitis cases by health district in 3 regions, Niger, 2002–2012; performance of epidemic threshold definitions in detecting elevated annual meningitis incidences at the health area and district levels, in a situation simulating elimination of meningococcal serogroup A meningitis in 3 regions, Niger, 2002–2012. [file 14-1361-Techapp-s1.pdf]

Article DOI: http://dx.doi.org/10.3201/eid2108.141361

Response Strategies against Meningitis Epidemics after Elimination of Serogroup A Meningococci, Niger

Technical Appendix

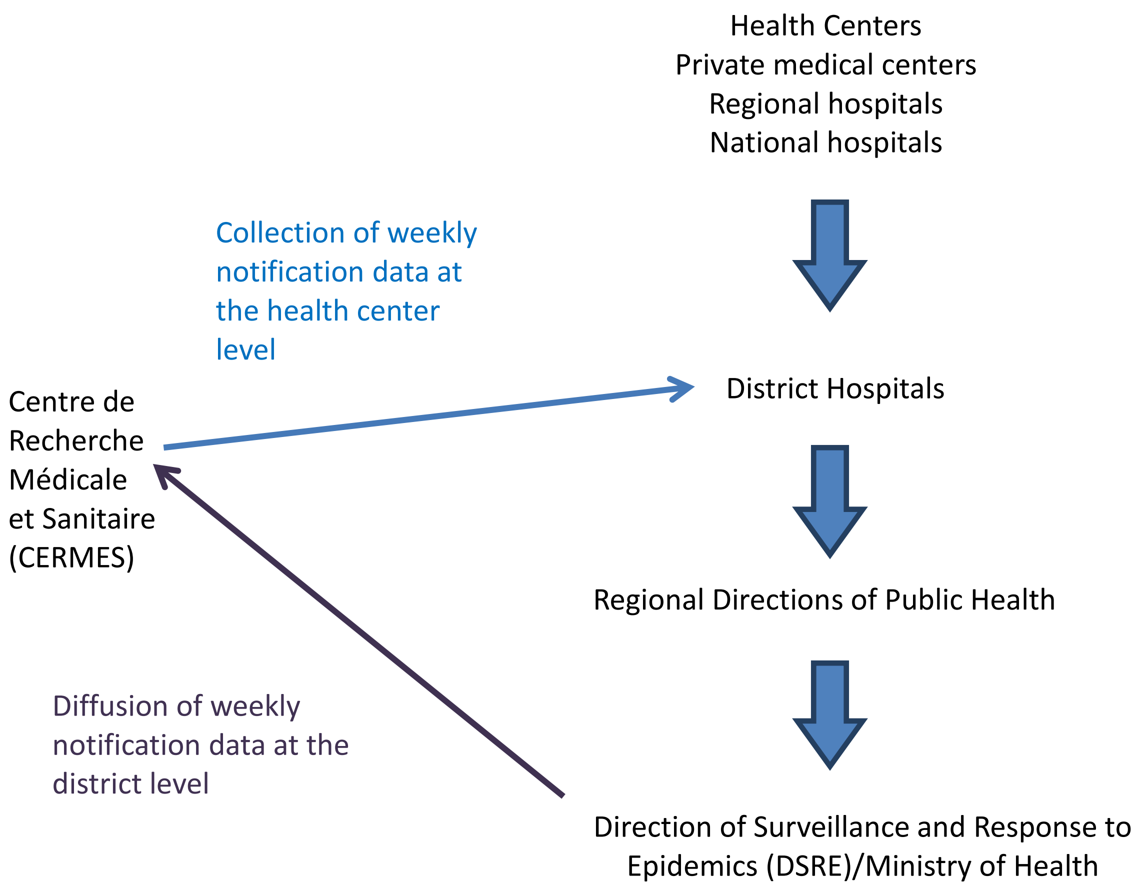

Technical Appendix Figure 1. Data transmission and collection for reporting of suspected meningitis cases, Niger. For national routine surveillance, all health facilities in Niger transmit weekly case counts to district hospitals, where data are aggregated to a district case count and transmitted to the Regional Directions of Public Health, then to the Direction of Surveillance and Response to Epidemics/Ministry of Health for reporting. To analyze epidemic dynamics at the health area level, we retrieved the original health facilities meningitis case counts at the district hospitals.

Evaluation of Completeness of the Health Center Database

The country has 8 regions (Tahoua, Tillabery, Agadez, Diffa, Maradi, Niamey, Zinder, and Dosso), 42 districts, and 732 health areas containing >1,500 health centers. To assess the completeness of this database, we compared the resulting district-level weekly case counts with those included in the national routine surveillance reports.

A ratio was calculated as the number of suspected meningitis cases in our database divided by the number of suspected meningitis cases in the national surveillance database. This ratio was calculated for every year (aggregated cases in our database by year/aggregated cases in the national surveillance database by year), then for every region (region-level aggregated cases in our database/region-level aggregated cases in the national surveillance database) and every district (district-level aggregated cases in our database/district-level aggregated cases in the national surveillance database). A ratio of 1 suggested no differences between the cases counts of the 2 databases. This ratio was ≈1 during 2008–2012, suggesting that our data from the whole country were almost complete for this period. At the region level, a ratio ≈1 was found for regions of Tahoua, Tillabery, Agadez, and Diffa. It was ≈0.78, 0.77, and 0.71, respectively, for Dosso, Maradi, and Zinder regions. Data for Niamey were not complete because the ratio was 0.10, so we excluded it. At the district level, we calculated the number of districts that have ratios less than the first quartile, between the first and the third quartiles, and more than the third quartile of all the ratios.

The first quartile of these ratios was 0.8, the median 1, and the third quartile ≈1.3. Over 70 district years, ratios were <0.8, 69% were from the regions of Maradi and Zinder, so we excluded them. Tahoua, Tillabery, and Dosso presented the highest number of district years (ratios 0.8–1.3).

We then calculated for each region the rate of missing health center years using data collected by the Centre de Recherche Médicale et Sanitaire. This operation showed that the Tahoua, Tillabery, Dosso, and Diffa regions had the lowest rate of missing data during the study period (<40%). Maradi and Zinder presented >40% of health centers as missing for 2002–2006. Agadez had >40% of missing health centers for 2002–2005 and 2012. Niamey also had >40% of missing data from 2002 to 2010. Thus, Niamey data were not usable, Maradi and Zinder could

be used only from 2007 to 2012 and Agadez from 2006 to 2011. Diffa did not bring important notified cases. Tahoua, Tillabery, and Dosso data were the most usable for the study period. With these analyses, we selected the regions of Tahoua, Tillabery and Dosso on the basis of 3 criteria: less difference in notified cases in the 2 databases, fewer missing data (<40%), and the number of notified cases.

A

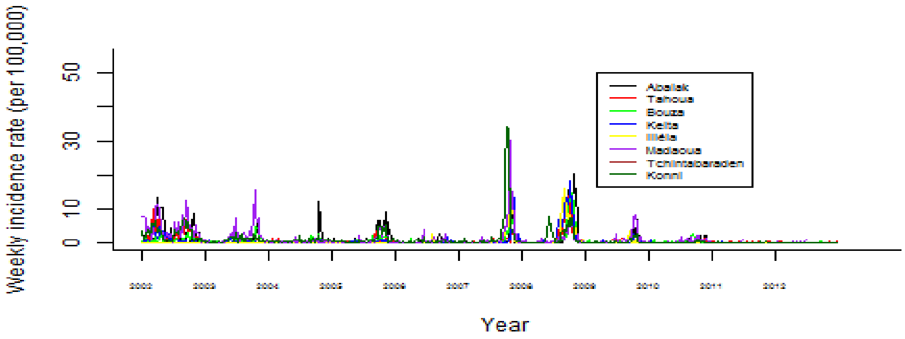

B

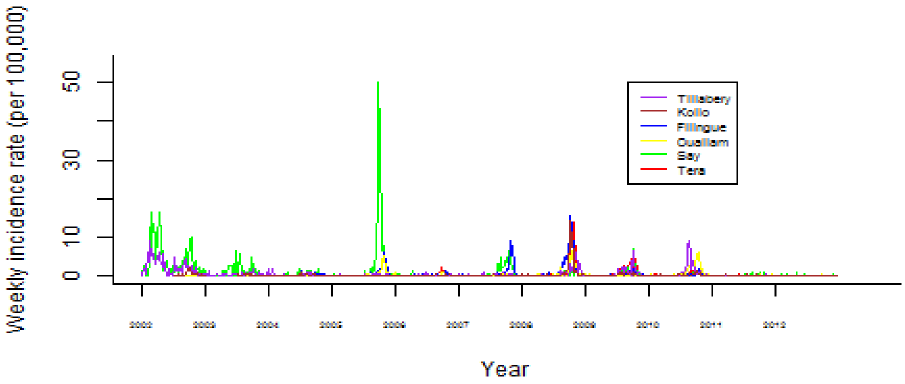

C

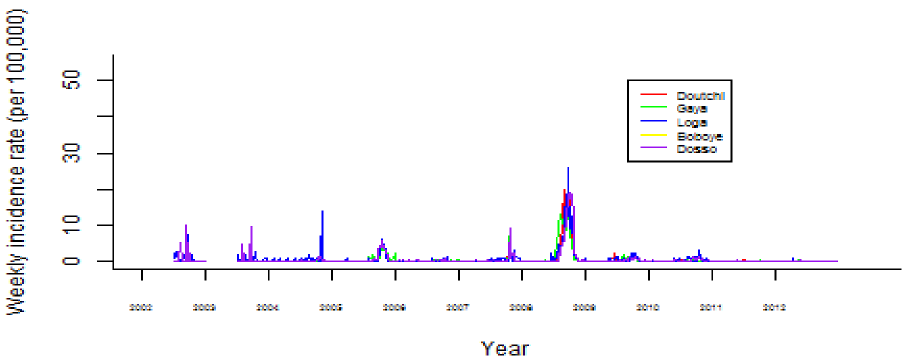

Technical Appendix Figure 2. Weekly incidence rates (WIR) of suspected meningitis cases by health district. Tahoua, Tillabery, and Dosso regions, Niger, 2002–2012. A) Tahoua region: The predominant etiology in the districts with peak WIR >10 cases per 100,000 inhabitants, where laboratory data were

available, was meningococcal serogroup A. B) Tillabery region: The predominant etiology in the districts with peak WIR >10 cases per 100,000 inhabitants, where laboratory data were available, was meningococcal serogroup X (Say). C) Dosso region: The predominant etiology in districts with peak WIR >10 cases per 100,000 inhabitants, where laboratory data were available, was meningococcal serogroup A (Gaya and Loga).

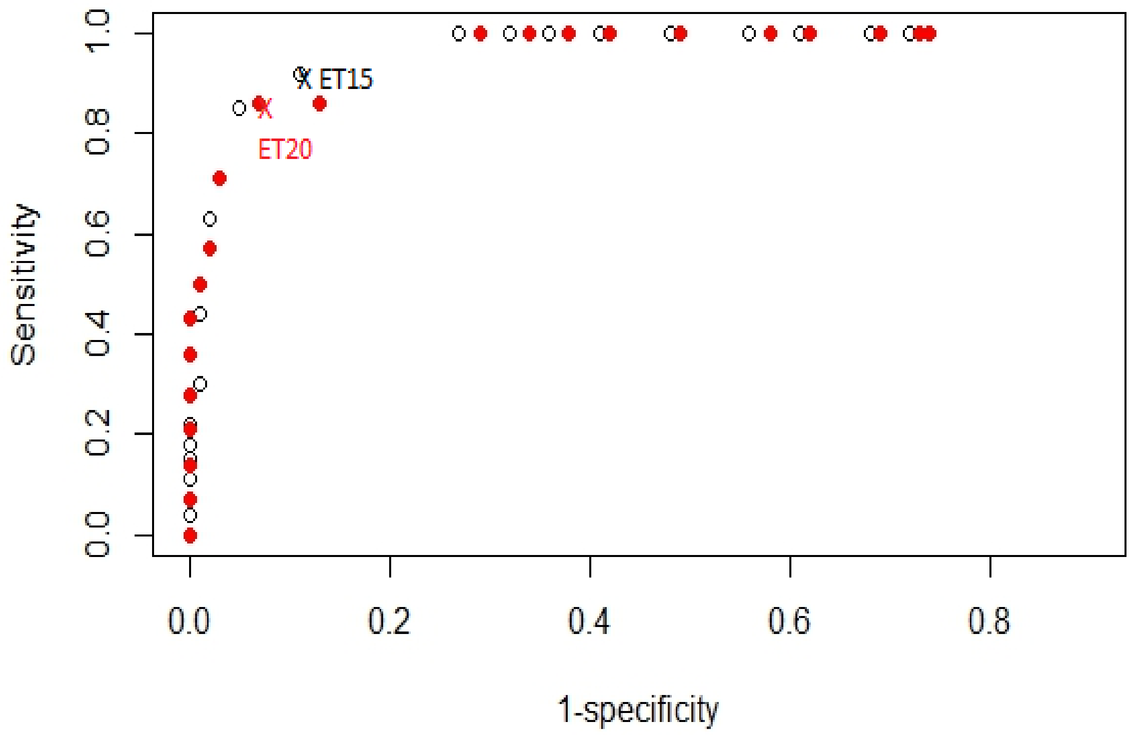

Technical Appendix Figure 3. Performance of epidemic threshold (ET) definitions in detecting elevated annual meningitis incidences at the health area level in a situation simulating elimination of meningococcal serogroup A meningitis (NmA). Tahoua, Tillabery, and Dosso regions, Niger, 2002–2012 (531 health area years)**.** All health area years with ≥1 NmA cases or without serogroup information were excluded. Empty marks represent the performance of ETs in detecting annual incidences ≥0.08% which corresponds to the 95th percentile of all annual incidences in the database. Full and red marks represent the performance in detecting annual incidences ≥0.12%, which corresponds to the 97.5th percentile of all annual incidences of the database. ETs were defined as weekly incidence rates of 1, 2, 3, 4, 5, 6, 7, 8, 9, 10, 15, 20, 30, 40, 50, 60, 70, 80, 90, and 100 cases per 100,000 inhabitants.

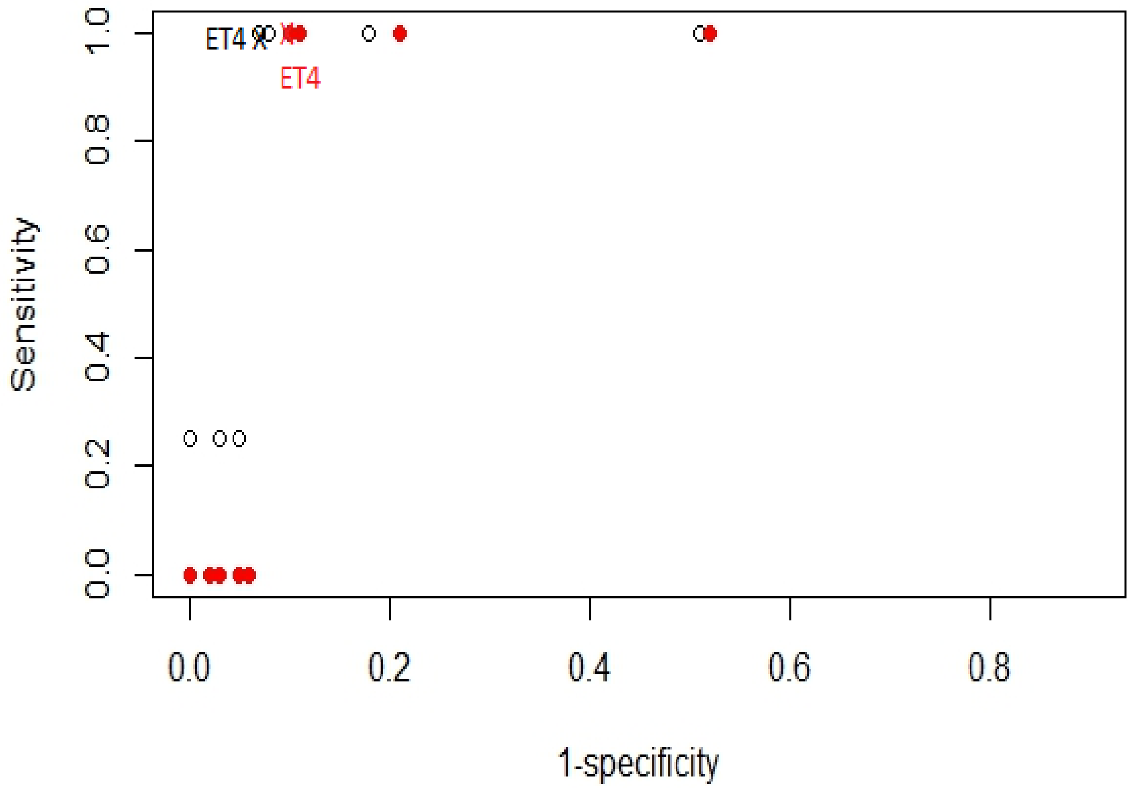

Technical Appendix Figure 4. Performance of epidemic threshold (ET) definitions in detecting elevated annual meningitis incidences at the district level, in a situation simulating elimination of serogroup A meningococcal meningitis, in Tahoua, Tillabery, and Dosso regions, Niger, 2002–2012 (65 district years)**.** All district years with ≥1 more NmA cases or without serogroup information were excluded. Empty marks represent the performance of ETs in detecting annual incidences ≥0.027% which corresponds to the 95th percentile of all annual incidences in the database. Full and red marks represent the performance in detecting annual incidences ≥0.029%, which corresponds to the 97.5th percentile of all annual incidences of the database. ETs were defined as weekly incidence rates of 1, 2, 3, 4, 5, 6, 7, 8, 9, 10, 15, 20, 30, 40, and 50 cases per 100,000 inhabitants.
